# Optimization of Polyphenols’ Recovery from Purple Corn Cobs Assisted by Infrared Technology and Use of Extracted Anthocyanins as a Natural Colorant in Pickled Turnip

**DOI:** 10.3390/molecules27165222

**Published:** 2022-08-16

**Authors:** Francisco J. Barba, Hiba N. Rajha, Espérance Debs, Anna-Maria Abi-Khattar, Stéphanie Khabbaz, Basharat Nabi Dar, Mario J. Simirgiotis, Juan Manuel Castagnini, Richard G. Maroun, Nicolas Louka

**Affiliations:** 1 Department of Preventive Medicine and Public Health, Food Science, Toxicology and Forensic Medicine, Faculty of Pharmacy, University of Valencia, 46100 Valencia, Spain; 2 Centre d’Analyses et de Recherche, Unité de Recherche Technologies et Valorisation Agro-Alimentaire, Faculté des Sciences, Université Saint-Joseph de Beyrouth, B.P. 17-5208 Riad El Solh, Beirut 1104 2020, Lebanon; 3 Ecole Supérieure d’Ingénieurs de Beyrouth (ESIB), Université Saint-Joseph de Beyrouth, CST Mkalles Mar Roukos, Beirut 1107 2050, Lebanon; 4 Department of Biology, Faculty of Arts and Sciences, University of Balamand, P.O. Box 100, Tripoli 1300, Lebanon; 5 Department of Food Technology, Islamic University of Science and Technology, Kashmir 192122, India; 6 Institute of Pharmacy, Faculty of Sciences, Universidad Austral de Chile, Elena Haverbeck S-N, Valdivia 5090000, Chile

**Keywords:** purple corn cobs, infrared-assisted extraction, response surface methodology, anthocyanins, natural colorant

## Abstract

An ecofriendly extraction technology using infrared (IR) irradiation *Ired-Irrad*^®^ was applied to purple corn cobs to enhance polyphenol recovery for the first time. The IR extraction efficiency was compared to that of the water bath (WB) method. Response surface methodology (RSM) using a central composite design was conducted to determine the effect of the experimental conditions (extraction time and treatment temperature) and their interactions on the total polyphenol and anthocyanin yields. Optimal extraction of total phenolic compounds (37 mg GAE/g DM) and total monomeric anthocyanins (14 mg C3G/g DM) were obtained at 63 °C for 77 min using IR as an extraction technique and water as a solvent. HPLC revealed that the recovery of peonidin 3-O-glucoside and cyanidin 3-O-glucoside was enhanced by 26% and 34%, respectively, when using IR. Finally, purple corn cobs’ spray-dried extract was proven to be an important natural colorant of pickled turnip. It offers great potential for use as a healthy alternative to the carcinogenic rhodamine B synthetic dye, which was banned.

## 1. Introduction

Maize (*Zea Mays* L.), or corn, is one of the most common staple crops. Hundreds of varieties, characterized by their diverse color shades (white, yellow, red, purple, etc.), are cultivated throughout the world. Purple corn is one of these genetic varieties and reigns in the Andean valleys [1,2]. It has a well-earned reputation for its remarkable richness in polyphenols, mainly anthocyanins [3], secondary metabolites that are essentially imparting their distinguished coloration to the corn. Although found in the grains, anthocyanins are abundant in the cobs of purple corn, which are generally considered as agro-industrial waste despite this richness [4,5]. Nowadays, valorization of any residual biomass is regarded to be fundamental to the circular economy. Hence, our ultimate aim is the valorization of purple corn cobs.

The anthocyanin extraction from the dark-colored corn cobs yields an intensely colored extract—a highly valuable natural colorant or dyeing agent [6] with multiple applications in many industries such as food, beverages, and cosmetics [7]. Being a subclass of flavonoids, anthocyanins are water-soluble pigments in the color range from red to blue to purple [8]. Furthermore, anthocyanins are interesting phenolic compounds that have shown potential anticancer activities [9], anti-inflammation effects [10], and platelet function control [11], among other important bioactivities [12,13]. The extracted pigment has been recognized by the European Union and Japan as a natural food colorant with the code E163 [14].

Anthocyanins are known to have low stability that seems to be affected by pH, temperature, oxygen, light, and other factors [15,16,17,18]. Consequently, it is crucial to consider a method that would extract the maximum amount of these bioactive compounds, while preserving their properties and real potential. Conventional methods using water [19] or other solvents [14,20] and emerging technologies such as ultrasounds [4], microwaves [21], or supercritical fluids [3] have proven to be efficient for the recovery of bioactive compounds from purple corn cobs. Each method has its advantages in terms of ease of implementation, low cost, solvent consumption economy, and extraction yield. Regarding this latter aspect, many other innovative techniques have been conceived for the intensification of polyphenol recovery from their corresponding matrices [22,23,24,25].

Extraction by *Ired-Irrad*^®^, a patented technology [26] based on the use of infrared irradiation, has already demonstrated its efficiency in terms of maximization polyphenol extraction. The IR energy, emitted as irradiation in the far wavelength range (3–4 μm up to 1000 μm), is used to heat the sample to be treated. To the best of our knowledge, no previous research has investigated the use of infrared irradiation on purple corn cobs. Therefore, the objective of the present study was to optimize the recovery of polyphenols from purple corn cobs using response surface methodology (RSM). The efficiency of the infrared technique (IR) in terms of yield, bioactivity, and composition of the extract will be compared to the conventional water bath extraction (WB). In the last part of the study, the extract obtained under optimal IR conditions was spray dried. The resulting powder was tested as a natural food colorant in pickled turnip in an attempt to replace the highly toxic synthetic dye rhodamine B.

## 2. Materials and Methods

### 2.1. Raw Material

Purple corn cobs (*Zea mays* L.) were supplied by Al-Kazzi industry. They were ground into finely powdered particles (Ephrem’s mills, Beirut, Lebanon), and separated using a vibrating multi-sieve separator (ELE international, Loveland, CO, USA). Based on a preliminary set of experiments, particles with a diameter between 850 µm and 2 mm were selected for experimentation and stored at 4 °C until further use.

### 2.2. Dry Matter

The dry matter content of purple corn cobs was measured by drying them in a ventilated oven at 105 °C until a constant weight was reached. The dry matter (DM) of initial raw material was 90 ± 0.2%

### 2.3. Extraction Procedures

#### 2.3.1. Water Bath-Assisted Extraction (WB)

The conventional extraction was carried out in a digital water bath (JSR JSWB-22T, Gongju-city, Korea). Based on a previous series of trials using a central composite design with similar experimental conditions as those shown in Table 1, and upon taking into consideration the total phenolic content (TPC) and total monomeric anthocyanin (TMA) yields, the optimal conditions for time (132 min), temperature (73 °C), and solid:liquid ratio of 1:70 (*w*:*w*) were defined. Water was adopted as the sole solvent used in this study.

#### 2.3.2. Infrared-Assisted Extraction Ired-Irrad^®^ (IR)

**Figure 1:**
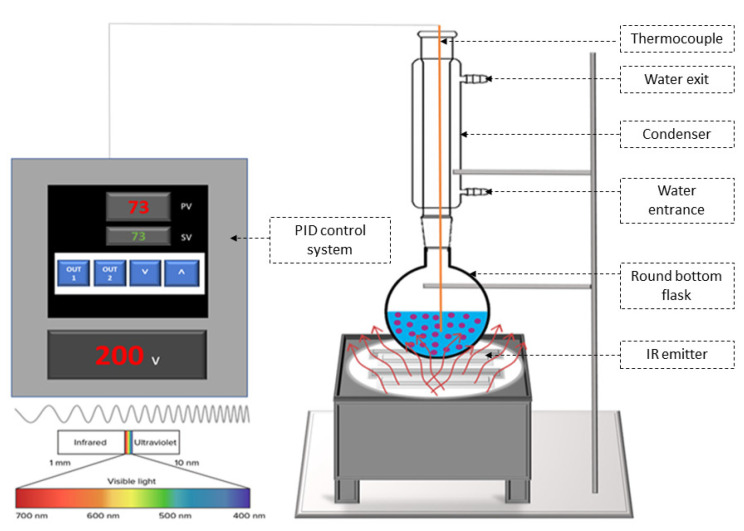
Infrared radiation instrument setup.

The basic principle of the apparatus (Figure 1) is to expose the plant matrix while being soaked in the solvent to the effect of infrared irradiation. Briefly, ground corn cobs mixed with water at a solid:liquid ratio of 1:70 *w:w* (1 g in 70 mL) were placed in a round-bottom flask at a distance of 1 cm from a black ceramic IR emitter (Rotfil, Pianezza, Italy). The flask is connected to a condenser to avoid solvent evaporation and modification of its volume. The IR emitter is equipped with a proportional–integral–derivative (PID) system in order to control the temperature of the sample that is recorded by a thermocouple type K (nickel–chromium/nickel–alumel) inserted within it. The experimental conditions, extraction times, and treatment temperatures were determined according to the experimental design described in the next paragraph.

### 2.4. Experimental Design

Optimization of the extraction of phenolic compounds by IR irradiation was conducted using an experimental design based on response surface methodology (RSM), which is a statistical approach employed for analyzing and empirically modeling a process. This methodology allows us not only to detect the significant effects (direct and quadratic) of the operating parameters and their interactions but also to quantify them. A rotatable central composite design (2^2^ + star) was created with five levels for the extraction time t—30, 52, 105, 158, and 180 min—and five levels for the treatment temperature T—25, 33, 52.5, 72, and 80 °C (Table 1). Twelve experimental trials were conceived, including four factorial points (−1 and +1), four repetitions at the center points (0,0), and four-star points (−α and +α) where α is 1.41. The central composite design was used to build a second-order model (quadratic) for the two response variables, which are the yields in total phenolic content and total monomeric anthocyanins.

### 2.5. Phenolic Compound Quantification

#### 2.5.1. Total Phenolic Content (TPC)

The concentration of total polyphenols in purple corn cobs was assessed by the Folin–Ciocalteu procedure. A volume of 0.2 mL of the extract was added to 1 mL Folin solution and 0.8 mL of sodium carbonate (75 g/L). Tubes were heated for 10 min at 60 °C then cooled in a refrigerator for the same period of time. The absorbance/optical density was assessed at 750 nm using a UV-Vis spectrophotometer (Biochrom Ltd., Cambridge, England). Total phenolic content was calculated as gallic acid equivalent (mg GAE/g DM) using a standard gallic acid curve [27].

#### 2.5.2. Total Monomeric Anthocyanins (TMA)

The pH differential method was applied to evaluate TMA [28]. The optical density was measured at a wavelength of 510 and 700 nm using a UV-Vis spectrophotometer (Biochrom Ltd., Cambridge, England). The anthocyanin content of purple cobs was calculated as the content of cyanidin-3-glucoside equivalents per gram of dry matter (mg C3G/g DM) by using a molar extinction coefficient of 26,900 L·cm^−1^·mg^−1^ and a molecular weight of 449.2 g/mol.

#### 2.5.3. High Performance Liquid Chromatography Analyses (HPLC)

The two techniques, IR and WB, were compared on the basis of their ability to extract peonidin 3-O-glucoside and cyanidin 3-O-glucoside from ground purple corn cobs. An HPLC apparatus (Knauer, Berlin, Germany) was used for analysis, using a Discovery HS C18, 5 μm, 250 × 4.6 mm, column (Supelco, Bellefonte, PA, USA) coupled to an HS C18, Supelguard Discovery, 20 × 4 mm, 5 μm, precolumn (Supelco, Bellefonte, PA, USA). The solvent system consisted of A—0.2% formic acid in water (*v/v*)—and B—69% methanol, 29% water, and 2% formic acid (*v/v/v*). The gradient employed was 100% A for 3 min, 90% A at 60 min, 60% A at 80 min, 40% A at 105 min, 20% A at 120 min, 0% A at 140 min, going back to 100% A and maintaining the initial conditions for column equilibration for 20 min. The detection was carried out at a wavelength of 520 nm [29].

### 2.6. Biological Activities of the Extracts

#### 2.6.1. Antioxidant Activity

Bleaching of the stable radical DPPH (2,2-diphenyl-1-picryl-hydrazyl) was employed. A total of 1.45 mL of DPPH solution were added to 50 µL of the extract. Trolox (positive control) was used as the standard for the dilution method. The optical density was measured at 515 nm after incubation for 30 min in the dark. Results were expressed as µg of Trolox equivalent per mL (µg TE/mL) [30].

#### 2.6.2. Cupric Reducing Antioxidant Capacity Assay

The cupric reducing antioxidant capacity (CUPRAC) was performed using an assay kit (Bioquochem, Asturias, Spain) according to the manufacturer’s protocol [31]. The antioxidant activity of the extract was measured by the oxidation of the copper (II)-neocuproine (2,9-dimethyl-1,10-phenanthroline). The sample (40 µL) was mixed with 200 µL of a previously prepared working solution and incubated for half an hour at room temperature. The absorbance was measured then at 450 nm and the results were expressed as mM Trolox equivalent (mM TE).

#### 2.6.3. Ferric Reducing Antioxidant Power Assay

The ferric ion reducing antioxidant power (FRAP) of purple corn cob extracts was determined using a standardized capacity kit (Bioquochem, Asturias, Spain) [31]. The reduction of the ferric complex by the extracts at an acidic pH was quantified. The diluted sample (10 µL) was mixed with 220 µL of FRAP working solution and left to react for 4 min. The absorbance was then measured using a microplate reader at 593 nm. The antioxidant activity was expressed as µM of iron(II) equivalent (µM iron(II)).

### 2.7. Spray Drying of the Extract and Its Use for Turnip Coloration

The purple corn cob extract obtained under optimal IR conditions was spray dried (Shanghai Attainpak, DC 1500 LSD, Shanghai, China) using the following operating conditions: airflow through the system, 0.7 L/h; feed rate, 600 mL/h; inlet temperature, 160 °C; and outlet temperature, 70 °C.

Two grams of the purple corn cob extract reduced into a red powder by spray drying were added to 500 g of sliced turnips sealed in a glass jar with 500 mL of brine/vinegar solution. The evolution of TMA concentration in the brine solution was monitored as a function of time in order to quantify the absorbed colorant quantity in the turnip slices.

### 2.8. Statistical Analysis

Experiments were conducted in triplicate. Means values and standard deviations were calculated. Treatment’s significance was evaluated by analysis of variance (ANOVA) and LSD tests (least significant difference) using Statgraphics^®^ Centurion XV (Statgraphics 18, The Plains, Virginia).

## 3. Results and Discussions

### 3.1. Optimization by RSM, the Time-Temperature Effect

RSM was used with the aim of studying the effect of the two independent variables of IR irradiation—extraction time t and treatment temperature T—in order to determine the optimal values required to maximize the yield in polyphenols (mg GAE/g DM) and anthocyanins (mg C3G/g DM) in the extracts. From this perspective, a central composite design with twelve runs was developed (Table 1). The obtained values of TPC and TMA in the IR extracts ranged between 15.77 and 38.65 mg GAE/g DM and between 7.33 and 13.04 mg C3G/g DM, respectively. For both responses, empirical models were statistically acceptable due to significant regression (*p* < 0.05) as well as non-significant lack of fit (*p* > 0.05). R^2^ was 99.3% and 98.6% for TPC and TMA, respectively. Equations (1) and (2) present the two models, as suggested by the experimental design, necessary to calculate the values of the two response variables TPC and TMA respectively:

TPC = −23.4 + 0.17t + 1.4T − 0.00038t^2^ − 0.0087T^2^ − 0.00089t × T (1)

TMA = −8.26 + 0.094t + 0.49T − 0.0003t^2^ − 0.0032T^2^ − 0.0004t × T (2)

**Figure 2:**
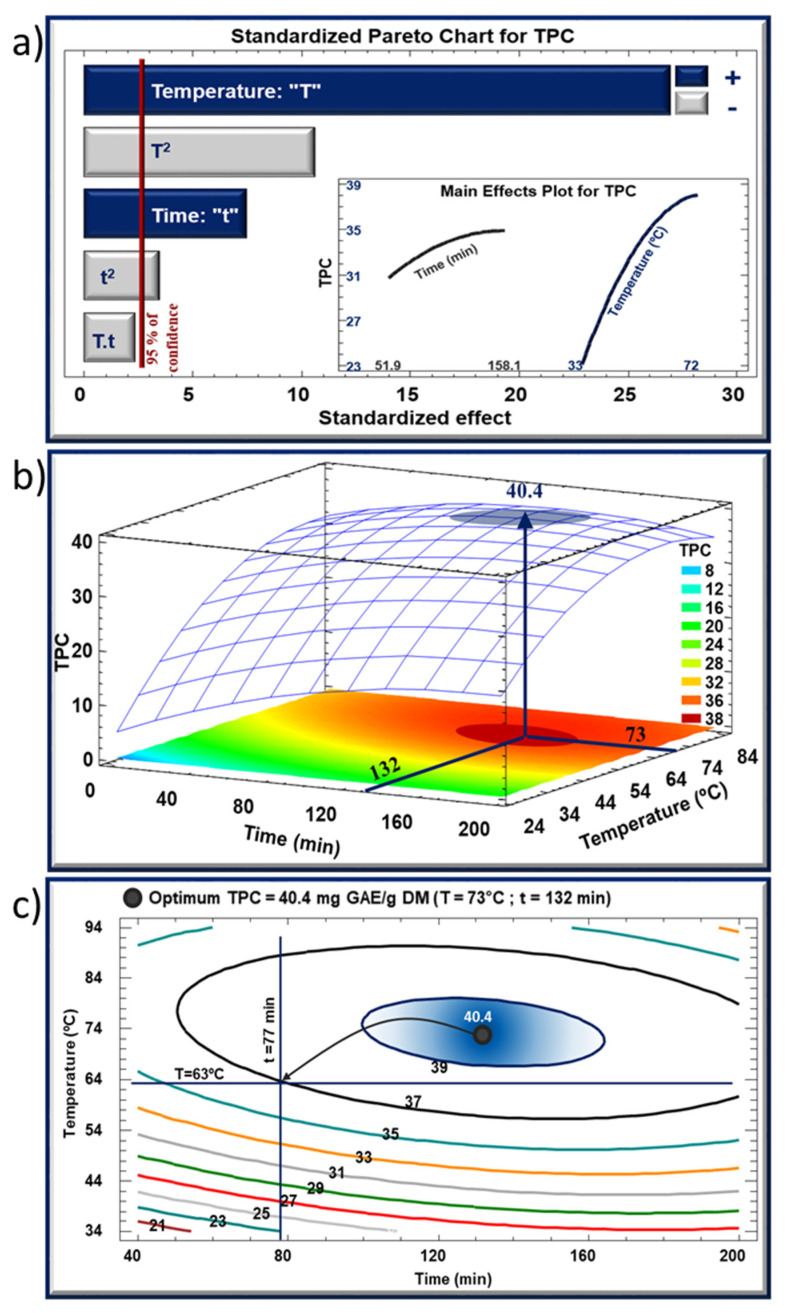
(**a**) Standardized Pareto chart, (**b**) the corresponding estimated response surface mesh of TPC, and (**c**) the corresponding estimated response contours for TPC recovery using IR-assisted extraction.

**Figure 3:**
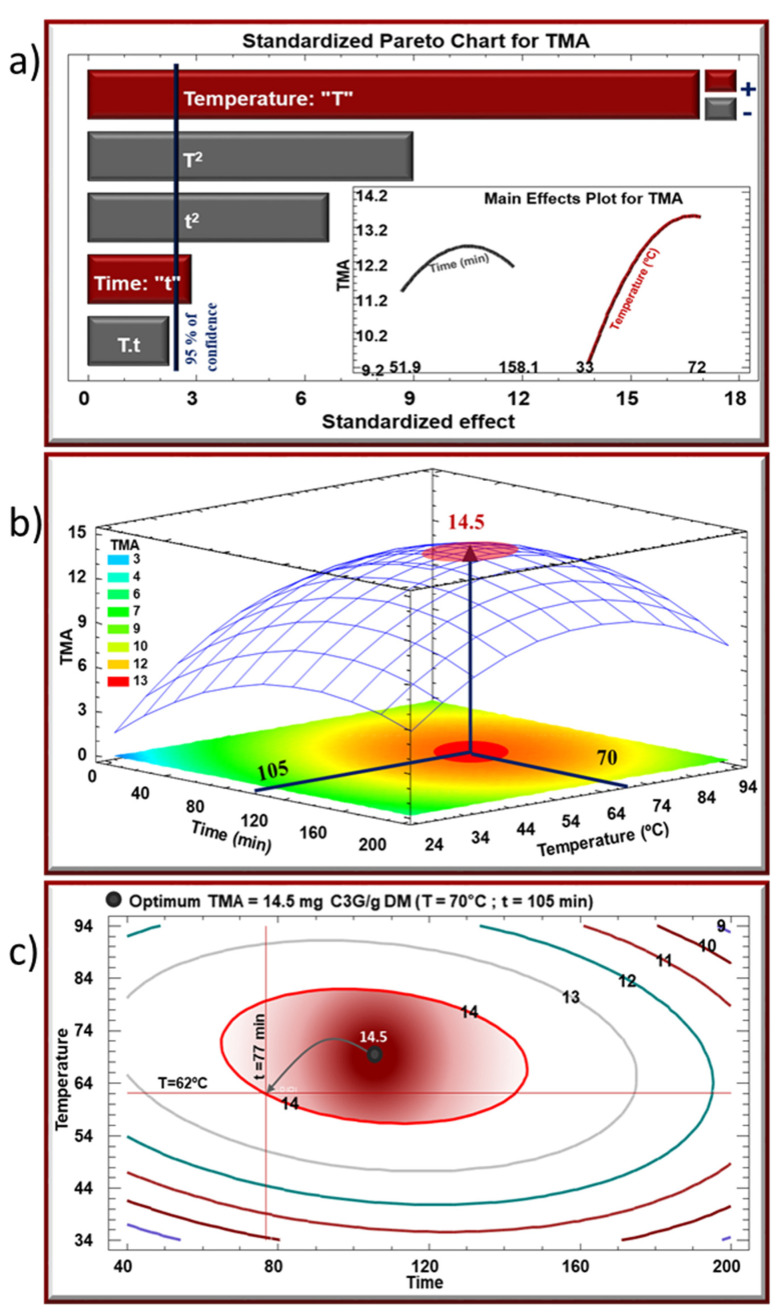
(**a**) Standardized Pareto chart, (**b**) the corresponding estimated response surface mesh of TMA, and (**c**) the corresponding estimated response contours for TMA recovery using IR-assisted extraction.

Figure 2a and Figure 3a depict the Pareto charts, where the bars are arranged in a descending order representing the significance level of the response variables. Bars that extend beyond the vertical line are considered statistically significant with more than a 95% confidence level. The two inserts display the evolution of TPC (2a) and TMA (3a) as a function of one parameter while the second parameter is maintained constant at its central value (center point 0). Although both parameters played a positive role in increasing the yield of TPC and TMA, treatment temperature T was much more influential. Meanwhile, quadratic effects of the two parameters (t^2^ and T^2^) were negative on both TPC and TMA yields. Indeed, the evolution of TPC and TMA yields as a function of T was characterized by a two-phase shape: a steep initial increase (positive linear effect) followed by a tendency towards stabilization (negative quadratic effect). This initial increase in TPC and TMA in the extracts suggests an enhanced affinity/solubility of these molecules in the solvent [14]. A convenient heating lowers the viscosity of the solvent leading to a higher diffusivity within the cells. As for the stabilization, a competition between two phenomena could account for this observation; the increase in temperature may have led to a release of larger amounts of polyphenols from the cells, yet such continuous increase may have had an alteration effect on a part of the released molecules.

On the other hand, changes in TPC and TMA yields as a function of t were much less marked. Increasing extraction time t led to a stabilization in TPC yield, although preceded by a slight increase (Figure 2a), whereas the same increase maximized the TMA yield that exhibited a subsequent decline phase. Here again, exposing the matrix to a relatively high temperature for a long period of time may have had opposing effects: a higher yield of extraction but a higher rate of degradation which prevailed in the case of TMA, thus explaining the decrease in the remaining amount of anthocyanins. Our results are in agreement with previous studies that demonstrated a similar pattern in the dissolution/extraction and instability/degradation rates of bioactive compounds with increasing temperature [32,33,34].

Regarding the interaction between the two parameters, no significant effect was observed for TPC or TMA. This observation means that the yields of TPC and TMA varied as a function of one parameter (t or T) regardless of the value of the second. On the other hand, and by referring to Table 1, one can observe that the two parameters exerted a synergistic effect on the yield of recovery. For instance, after an increase from −1 level to +1 level, treatment time (comparison between run 1 and run 2, and between run 3 and run 4) and extraction temperature (comparison between run 1 and run 3, and between run 2 and run 4) provoked an increase in TPC yield by about 4 mg GAE/g DM and 15 mg GAE/g DM on average, respectively. Nonetheless, a simultaneous increase in t and T from their −1 level to +1 level (comparison between run 1 and run 4) increased TPC by approximately 19 mg GAE/g DM suggesting that the effects of the two parameters was additive.

Figure 2b and Figure 3b showcase the response surfaces of TPC and TMA respectively as a function of t and T simultaneously. These three-dimensional models are representations that inform the optimal zones (in this case maximal values) of TPC and TMA. In these figures, optimal values of 40.4 mg GAE/g DM for TPC and 14.5 mg C3G/g DM for TMA would be obtained at 73 °C for 132 min and 70 °C for 105 min, respectively. Projection of the response surface profiles onto the bottom of the graph results in a spectrum of color gradients ranging from blue (lowest level) to red (highest level). Figure 2c and Figure 3c show the isoresponse contour plots presented as a series of concentric ellipses around the optimal zones, and the numbers indicated on every contour correspond to values of TPC and TMA in Figure 2c and Figure 3c, respectively. For instance, the contour tagged with number 37 in Figure 2c designates all pairs of time/temperature that are required to obtain a TPC value of 37 mg GAE/g DM, which allows a greater scope in the choice of operating parameters in order to optimize the whole operation cost. This way, it would be possible to select a TPC value of 37 mg GAE/g DM instead of the maximal value (40.4 mg GAE/g DM), thus saving extraction time (77 min, instead of 132 min) and reducing treatment temperature (63 °C, instead of 73 °C). Similarly, in Figure 3c, a TMA value of 14 mg C3G/g DM instead of 14.5 mg C3G/g DM would spare 28 min and decrease the temperature by 8 degrees.

**Figure 4:**
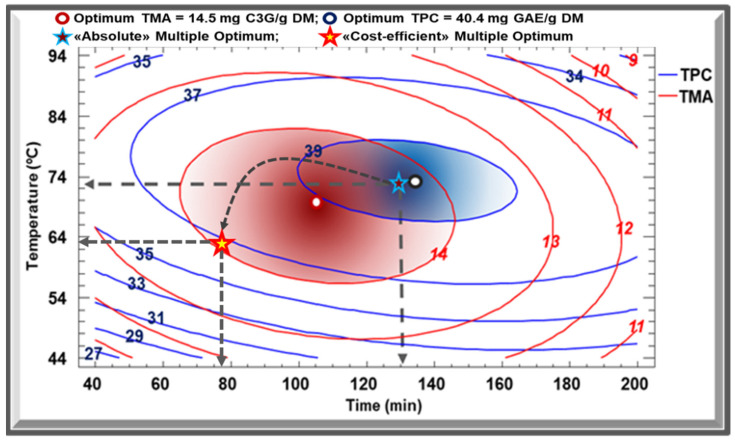
Overlap of TPC and TMA contour plots. The blue and red ellipses correspond to TPC and TMA optimal zones respectively.

When combining TPC and TMA contour plots, optimal zones partially overlap. In Figure 4, the two ellipses blue and red corresponding to TPC and TMA optimal windows respectively, have a common area that allows the selection of a multiple optimum. A compromise between the two experimental parameters can be found to achieve a product with the best overall acceptability. Having said that, an “absolute” multiple optimum, designated by the blue star in Figure 4, is suggested by the model so that it corresponds to 40.37 mg GAE/g DM and 14.2 mg C3G/g DM of TPC and TMA, respectively. However, and after considering the two optimums in Figure 2c and Figure 3c, another multiple optimum, a less costly one, can be chosen. Designated by the red star, this “cost-efficient” multiple optimum corresponds to a slight decrease in TPC and TMA yields (37 mg GAE/g DM and 14 mg C3G/g DM, respectively), yet could be achieved with more economical operating conditions: 77 min at 63 °C. By practically using and applying the latter conditions, the obtained TPC and TMA yields confirmed and validated the predicted values.

Extraction using IR yielded better TPC and TMA contents than the optimal values obtained with conventional water bath extraction, which were 32 mg GAE/g DM and 11.5 mg C3G/g DM, respectively. Moreover, the optimal IR conditions reduced the duration of the process by 1.7 times and the temperature by 10 °C, which is likely to affect the overall energy consumption of the process compared to the optimal water bath conditions (132 min and 73 °C).

Referring to the literature, the anthocyanin content of the purple corn cobs in the optimal water bath conditions reported in this study (11.5 mg C3G/g DM) is a little bit lower than that reported using a conventional solid–liquid extraction in a water–ethanol ratio (around 1:1), that yielded a monomeric anthocyanin content of >13.5 mg/g FW [14]. This lower yield is probably due to the extraction solvent, which was only water in our study. Moreover, the obtained yield (11.5 mg C3G/g DM) was also lower than that recovered using solid–liquid extraction with b-cyclodextrin addition to the water solvent. With the addition of 0.1 mg/mL of β-CD to water at 52 °C during 105 min, 12.32 mg C3G/g DM were recovered, whereas 24.97 mg C3G/g DM were obtained with a concentration of 49.8 mg/mL of β-CD in water. β-CD was shown to enhance polyphenol and anthocyanin recovery from purple corn cobs [35].

On the other hand, innovative technologies were also used to recover polyphenols from purple corn cobs. A supercritical carbon dioxide extraction (400 bars, 50 °C) of polyphenols from purple corn cobs was performed using a 70% ethanol/water mixture as a co-solvent, and yielded a high polyphenol concentration of 290 mg GAE/g and an anthocyanin content of 66 mg C3G/g dried extract with an antioxidant activity of 31 μg/mL, as determined by DPPH. Three major anthocyanins, cyanidin-3-glucoside, peonidin-3-glucoside, and pelargonidin-3-glicoside, were identified in the extracts [36]. Supercritical solvent technique yielded almost 7 times higher TPC and 4.6 times higher TMA than those obtained in this study using IR in the optimized conditions.

Moreover, the optimization of ultrasound-assisted (US) extraction of TMA and TPC from dried purple waxy corn cobs was also investigated. Optimal conditions (water–ethanol ratio 1:1, amplitude level of US 50%, 65 °C, 30 min, solid–liquid ratio 1:20) allowed the obtainment of 0.24 mg C3G/g DM and 27.662 mg GAE/g DM, with an antioxidant activity of 4.64 mg/mL. Eight anthocyanins were identified: cyanidin-3-glucoside, pelargonidin-3-glucoside, peonidin-3-glucoside, cyanidin-3-(6-malonylglucoside), pelargonidin-3-(6-malonylglucoside), peonidin-3-(6-malonylglucoside), cyanidin-3-(6-ethylmalonylglucoside), and petunidin-3-glucoside [4]. The IR results obtained in this study were almost 1.5 times and 60 times higher for TPC and TMA, respectively.

Considering the multiple varieties of the used raw materials in the different studies, the dissimilarities in their pretreatment processes (drying, storage time, particle size, grinding process), as well as variations in the operating conditions (time, temperature, solvent type, solid-to-liquid ratio, etc.), it would not be possible to draw a sound conclusion by comparing the efficiencies of the different extraction methods.

### 3.2. Antiradical Activity

**Figure 5:**
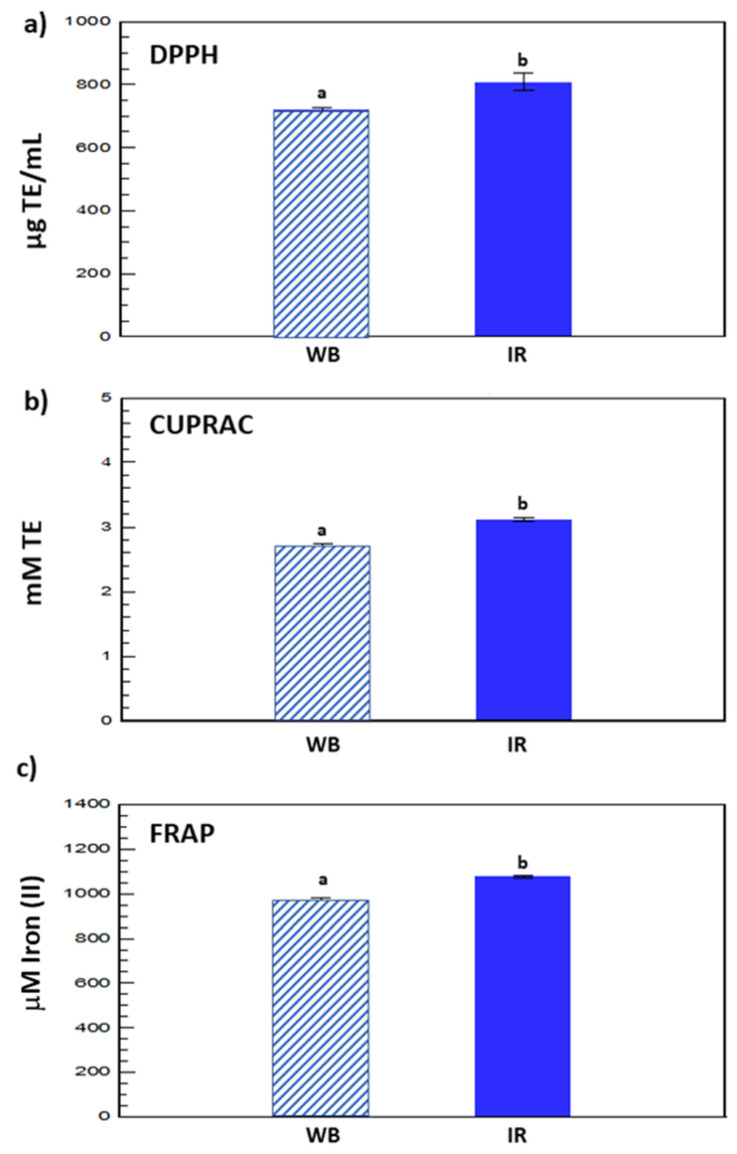
DPPH (**a**), CUPRAC (**b**), and FRAP (**c**) analyses of the WB and the IR extracts. Different letters (a,b) indicate signficant differences.

In order to evaluate the in vitro antioxidant capacity of purple corn cob extracts, DPPH, CUPRAC, and FRAP assays were applied. The assays were used to measure and compare the ability of the two extracts obtained under optimal conditions by WB (see paragraph 2.3.1) and IR (see the previous paragraph), to scavenge free radicals. As depicted in Figure 5, the IR extract exhibited a higher antioxidant potential when compared to the WB extract. Results of DPPH, CUPRAC, and FRAP were 807.96 ± 28.14 µg TE/mL, 3.11 ± 0.026 mM TE, and 1078.16 ± 5.89 µM iron(II) against 718.74 ± 8.58 µg TE/mL, 2.71 ± 0.039 mM TE, and 973.16 ± 10.60 µM iron(II) for IR and WB extracts, respectively.

The antioxidant activity of the cob extracts could be associated with their high total phenolic content. Indeed, polyphenols are one of the most effective antioxidative constituents in the plant [20]. Furthermore, a linear positive correlation was observed between phenolic content and antiradical activity, which highlights the usefulness of purple corn cob extracts for enhancing the oxidative stability of foods. Accordingly, purple corn cob extracts could potentially be suggested to prevent oxidative damage in biological systems due to their ability to scavenge oxygen species such as hydroxyl radicals.

### 3.3. High-Performance Liquid Chromatography

**Figure 6:**
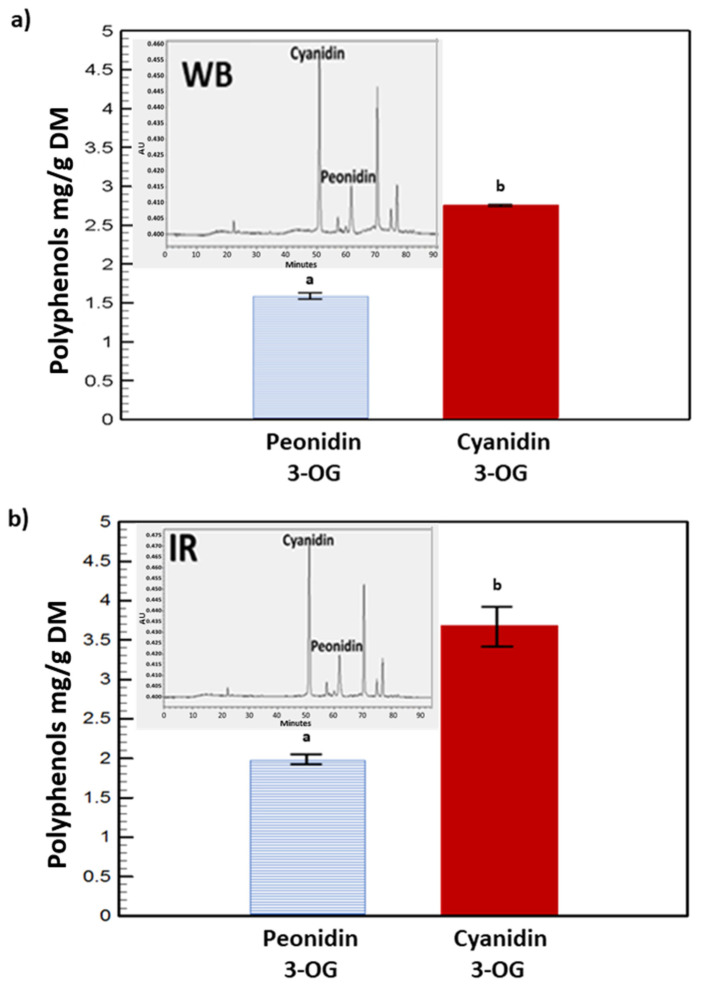
HPLC analyses of peonidin 3-O-glucoside and cyanidin 3-O-glucoside in WB (**a**) and the IR (**b**) extracts. Different letters (a,b) indicate signficant differences.

Peonidin 3-O-glucoside and cyanidin 3-O-glucoside are major anthocyanins identified in purple corn cobs extracts [19]. These two bioactive compounds were specifically selected as indicators of the quality of the recovered extracts. As indicated by the HPLC results in Figure 6, the use of the IR technique led to a higher extraction yield of peonidin 3-O-glucoside and cyanidin 3-O-glucoside from purple corn cobs by 26% and 34% respectively, when compared to WB. With a *p*-value less than 0.05, the statistical significance of the latter results was validated. The effectiveness of IR irradiation is highly attributable to the nature of the waveband released from an infrared source. Such electromagnetic radiation emitted continuously in the far wavelength range is known to have a high penetration power [33]. Furthermore, infrared waves offer a unique heating mechanism, resulting in higher extraction efficiency. In contrast to conventional heating whereby the heated solvent acts on the cell surface by convection, infrared energy can be absorbed by the solvent (water in our case) and can penetrate the cell surface and act on the constituents of the inner matrix [37]. These phenomena will enhance molecular vibrations in various manners, such as stretching, bending, rocking, and twisting within the plant matrix [37,38]. A higher molecular motion will, on one hand, promote the entry of the extracting solvent within the cellular structure and solubilization of the target compounds and, on the other hand, the exudation of cellular constituents, thus favoring extraction and improving the yield.

### 3.4. Use of Anthocyanins as an Antioxidant Colorant

**Figure 7:**
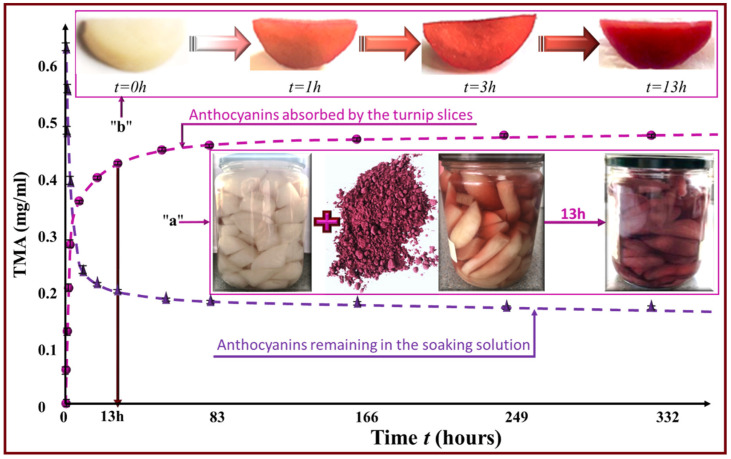
Evolution of TMA concentrations remaining in the soaking solution (descending curve) and absorbed by the turnip slices (ascending curve).

Extract of purple corn cobs was used and assessed as a colorant of pickled turnips. TMA contents of the brine solution and turnip slices were monitored by continuous sampling as a function of time. Figure 7 illustrates the evolution of TMA remaining in the soaking solution (descending curve) as well as that of TMA absorbed by the turnip slices (ascending curve). TMA reached a stationary concentration in both media after about 13 h of soaking. The insert 7a shows a jar of sliced turnip before adding the extract, immediately after adding the extract, and 13 h later. As for insert 7b, it reveals the color evolution in one slice of turnip after 1 h, 3 h, and 13 h of soaking in the extract-colored brine solution.

It can be concluded that purple corn cob extracts for dyeing pickled turnip has great application potential. The colorant is natural and is known for its antioxidative activity. It can be considered as a perfect alternative for the synthetic dye rhodamine B, which is most often used due to its high stability. Nevertheless, rhodamine B is listed among the internationally banned colorants since it is a highly carcinogenic compound and could cause liver malfunction [39]. Despite this fact, some Lebanese commercial brands were found to be non-compliant with international standards due to the addition of rhodamine B in order to color pickled turnips [40]. Our results may provide a solution for food companies that are interested in replacing synthetic dyes with natural colorants such as purple corn cob extract that is environmentally and consumer friendly. The naturally colored food is likely to acquire, besides the aesthetic aspect, an ameliorated health-beneficial effect thanks to the biological effects of the absorbed anthocyanins. Finally, it is noteworthy to mention that anthocyanins are the most stable at very low pH values [41].

## 4. Conclusions

The interest of the agro-industrial sector in recovering bioactive compounds from plant wastes has been tremendously increasing. When applying appropriate and efficient technologies, phenolic compound extraction from purple corn cobs could be a valuable way to valorize by-products generated by the corn industry. The present work investigated the influence of IR experimental conditions (time and temperature) on the extraction yield of TPC and TMA. The optimal extraction efficiency and antioxidant capacity results were obtained after 77 min at 63 °C. Under these conditions, the simultaneous optimization of TPC and TMA extraction provided a concentration of 37 mg GAE/g DM and 14 mg C3G/g DM, respectively. Extraction using infrared was faster than the water bath method, with a reduced extraction time, and more efficient in providing higher amounts of TPC and TMA in the extracts. Two major anthocyanins, peonidin 3-O-glucoside and cyanidin 3-O-glucoside, showed significant increases in the IR extracts of 26% and 34%, respectively, in comparison with water bath extraction.

Purple corn cob extract proved to be effective when used as a natural colorant of pickled turnips instead of synthetic artificial dyes, which offers the possibility to extend this practice to other industrial applications. Further analyses would be required to increase the stability and the bioavailability of anthocyanins contained in the extracts. *Ired-Irrad***^®^** is an environmentally friendly technology and is easy to handle, with a low manufacturing cost. It can be presented as an appropriate procedure for the intensification of polyphenol recovery from corresponding plant materials.

## Figures and Tables

**Table 1:** TPC and TMA values obtained according to the response surface experimental design by infrared-assisted extraction.

Trials	Time Coded Value (t) (Min)		Temperature Coded Value (T) (°C)		TPC (mg GAE/g DM)	TMA (mg C3G/g DM)
	Coded Variables	Decoded Variables	Coded Variables	Decoded Variables		
1	−1	52	−1	33	19.77	7.37
2	+1	158	−1	33	25.84	9.26
3	−1	52	+1	72	36.27	12.7
4	+1	158	+1	72	38.65	13.04
5	−α	30	0	52.5	28.18	10.48
6	+α	180	0	52.5	33.93	10.88
7	0	105	−α	25	15.77	7.33
8	0	105	+α	80	37.44	12.76
9	0	105	0	52.5	33.93	12.58
10	0	105	0	52.5	33.62	12.66
11	0	105	0	52.5	33.89	12.56
12	0	105	0	52.5	33.98	12.67

## Data Availability

Not applicable.
